# Mechanistic advances in neutrophil extracellular traps promoting tumor invasion via the PAR2 signaling axis

**DOI:** 10.3389/fimmu.2026.1983115

**Published:** 2026-10-21

**Authors:** Hongwei Xu, Wei Zhang

**Affiliations:** 1 Department of Urology, The Second Affiliated Hospital of Zunyi Medical University, Zunyi, Guizhou, China; 2 Department of Medical Records, The Second Affiliated Hospital of Zunyi Medical University, Zunyi, Guizhou, China

**Keywords:** epithelial-mesenchymal transition (EMT), neutrophil extracellular traps (NETs), protease-activated receptor 2 (PAR2), signal transduction, tumor invasion, tumor microenvironment

## Abstract

Neutrophil extracellular traps (NETs), long recognized as effectors of innate host defense, are now firmly implicated in remodeling the tumor microenvironment and facilitating tumor progression. Tumor-associated NETs expose proteases, damage-associated molecular patterns, and self-DNA that activate protease-activated receptor 2 (PAR2) on tumor and stromal cells, engaging downstream cascades (Gαq/11-PLCβ-PKC-MEK-ERK, RhoA-ROCK, β-arrestin-biased signaling, and RTK transactivation), which jointly drive epithelial-mesenchymal transition, matrix degradation, and angiogenesis, ultimately favoring invasion and metastasis. Direct evidence that NET-derived proteases engage PAR2 on tumor or stromal cells rests, however, on a limited number of studies, and much of the literature concerns NETs or PAR2 individually rather than their coupling. We searched PubMed with structured queries combining “neutrophil extracellular traps”/”NETs”, “protease-activated receptor 2”/”PAR2”/”F2RL1”, and cancer-related descriptors, supplemented by citation tracing, with coverage through August 2026, and graded each mechanistic claim at claim level as strong, moderate, or emerging according to explicit criteria (see Literature search and evidence grading); findings supported for NETs or PAR2 alone are labeled as such throughout. The axis is documented most completely in breast, pancreatic, lung, and colorectal cancers, where tumor-type-specific drivers have been defined (dormant-cell reawakening in breast cancer, stromal reprogramming in pancreatic cancer, bypass of targeted therapy in lung cancer, and bidirectional signaling in colorectal cancer), whereas in biliary and cervical cancers only descriptive NET data are available; therapeutic strategies targeting the axis (PAD4 inhibitors, DNase I, PAR2 antagonists/antibodies, and combinations with immune checkpoint blockade) show preclinical promise. However, validated biomarkers of pathway activity and safety data for chronic PAR2 blockade are lacking. The NETs-PAR2 axis is an emerging targetable driver of tumor invasion, and biomarker-guided, tumor-type-aware clinical development is required to translate axis-directed therapy into the clinic.

## 1 Introduction

Neutrophil extracellular traps (NETs) are web-like structures built from decondensed chromatin DNA decorated with histones, neutrophil elastase (NE), myeloperoxidase (MPO), cathepsin G, and matrix metalloproteinases such as MMP9. They are expelled by activated neutrophils, either through lytic cell death or from viable cells, and were initially characterized as antimicrobial structures (1, 2). Over the past two decades, NETs have been implicated in autoimmune disease, thrombosis, sepsis, and cancer, where they promote progression, metastasis, and immune evasion (3–6).

In the tumor microenvironment (TME), neutrophils are recruited and activated by hypoxia, inflammatory cytokines, and tumor-derived chemokines such as CXCL8 and CXCL2. NET density in tumor tissue correlates with advanced stage and poor prognosis, positioning NETs as mediators that couple inflammation to cancer progression (6, 7). Functionally, NETs stimulate tumor cell invasion and migration, promote epithelial-mesenchymal transition (EMT), support angiogenesis, and establish an immunosuppressive milieu that shields tumor cells from cytotoxic lymphocytes; they also interact with circulating tumor cells (CTCs), aiding their adhesion and extravasation at distant sites (6, 8).

A central molecular route for these effects is protease-activated receptor 2 (PAR2), a G protein-coupled receptor expressed on tumor cells, cancer-associated fibroblasts, endothelial cells, and macrophages. PAR2 is activated when serine proteases cleave its N-terminal domain to expose a tethered ligand; NET-associated proteases such as NE and proteinase 3 (PR3), as well as coagulation proteases including factor Xa and the tissue factor (TF)-FVIIa complex, can perform this cleavage (9, 10). In breast cancer cells, NETs induce TF expression and the TF/PAR2 axis mediates much of their pro-tumorigenic effect (9).

Therapeutically, inhibiting NET formation or PAR2 signaling has shown promise in preclinical models: PAD4 inhibitors reduce NET burden and tumor progression, PAR2 antagonists blunt the pro-invasive cascades initiated by NET-associated proteases (6, 11), NET clearance sensitizes resistant tumors to immune checkpoint blockade (12), and PAR2 blockade has been proposed as an adjunct to tyrosine kinase inhibitors in EGFR-mutant lung cancer (13). What remains unresolved is which molecular switches govern the NETs-PAR2 interaction and whether pathway blockade interferes with immune checkpoint therapy, a delicate point because PAR2 can, in some contexts, support antitumor immunity (14).

These questions frame the present review, which differs from previous surveys of NETs in cancer in three ways: it is organized around PAR2 rather than NETs in general as the central signaling node; it grades each mechanistic claim as strong, moderate, or emerging according to explicit criteria; and it treats tumor-type heterogeneity as the central biological insight (**Table 1**) that will determine which patients benefit from which intervention. Literature was identified through structured PubMed searches combining “neutrophil extracellular traps”/”NETs”, “protease-activated receptor 2”/”PAR2”/”F2RL1”, and cancer-related descriptors, supplemented by citation tracing from key mechanistic studies; coverage extends through August 2026.

**Table 1:** Evidence matrix for the NETs-PAR2 signaling axis across tumor types.

Tumor type	*In vitro* evidence	*In vivo* evidence	Clinical evidence	Overall appraisal
Breast (TNBC)	NETs induce TF expression and TF/PAR2-dependent pro-tumor gene expression in cancer cells (9)	PAD4 inhibition and DNase I reduce metastasis (8, 15); NETs awaken dormant cancer cells (16, 17); circulating NETs sequester tumor cells (6)	No direct clinical evidence of NET-PAR2 coupling; a NET-related prognostic signature includes F2RL2, which encodes PAR3 rather than PAR2 (18)	Strong (preclinical); the most complete NETs-PAR2 chain of evidence, but derived almost exclusively from TNBC/basal-like models, and without direct clinical evidence
Pancreatic (PDAC)	PAR2 activation of stellate cells drives collagen/fibronectin deposition and FAK-dependent collective invasion (19, 20)	NET-driven stromal remodeling and chemoresistance axes (TIMP1-CD63, GPRC5A-CXCL8) (21, 22); DNase I restores gemcitabine sensitivity (23)	Emerging; descriptive NET burden correlations only	Moderate; mechanistically strong but the PAR2 step is often inferred rather than directly tested; drug delivery barrier unresolved
Lung (NSCLC)	PAR2 blockade reverses osimertinib resistance via ERK-EMT and PD-L1 downregulation (13)	N2 neutrophil polarization maintains NETosis and immune suppression (24)	Smoking-associated NET-PAR2-ERK activation documented in tumor tissue (24)	Moderate; direct PAR2 intervention data limited to TKI-resistance models
Colorectal	PAR2 enhances tumor-cell adhesion to liver sinusoidal endothelium; NE-mediated PAR2 inactivation downregulates CD24 (11)	Bidirectional effects: NETs support hepatic metastatic niche formation, yet the NE-CD24 axis inhibits metastasis (11)	NET-related gene signatures prognostic in CRC and liver metastases (25, 26)	Moderate and complex; the axis is bidirectional; the dominant direction is context-defined
Cholangiocarcinoma/biliary tract	NET scaffold promotes progression via ITGAV/NF-κB (27)	Scaffold-driven angiogenesis and progression; high NETs score predicts poor survival (27)	Descriptive NET burden and prognosis only; PAR2 involvement not demonstrated in situ	Emerging
Cervical	S100A7-driven NET formation (28)	NETs facilitate lymph node metastasis (28)	Descriptive	Emerging

Literature search and evidence grading. PubMed was searched with the query pattern (“neutrophil extracellular traps” OR “NETs”) AND (“protease-activated receptor 2” OR “PAR2” OR “F2RL1”) AND (cancer OR tumor OR carcinoma OR metastasis OR invasion), updated through August 2026, and supplemented by citation tracing from key mechanistic studies. Studies were included if they reported original data on NET formation, PAR2 signaling, or NET-PAR2 coupling in tumor or relevant stromal contexts; peer-reviewed mechanistic reviews were used for background and synthesis; unreviewed conference abstracts were excluded. Each mechanistic claim was graded by the authors as follows: strong, the study directly demonstrates both a NET component and PAR2 activation or dependence (for example, protease cleavage of PAR2, or rescue of a NET phenotype by PAR2 loss-of-function); moderate, NET effects and PAR2 effects are separately demonstrated and converging evidence links them, but direct engagement is not shown; emerging, descriptive or correlative evidence only. Claims are tagged at claim level in Sections 3–7 and in **Table 1**. Where evidence supports NETs or PAR2 individually but not their coupling, we refer to NETs or PAR2 alone rather than using the compound term “NETs-PAR2 axis”. This three-tier scheme is a communication heuristic intended to make the strength of evidence legible to readers; it is not a formal quality-assessment instrument such as GRADE.

## 2 Biological characteristics and formation mechanisms of NETs

### 2.1 Structure and composition of NETs and their classification

NETs consist of a chromatin DNA backbone decorated with more than 30 protein components, including the antimicrobial peptide LL-37, granular enzymes such as NE, MPO, and cathepsin G, and citrullinated histones H3 and H4 (1, 2). The exact protein payload of a NET is stimulus- and context-dependent: NETs generated by tumor-derived signals differ in composition from those induced by bacteria, and these differences translate into distinct effects on neighboring cells. **Figure 1** outlines the composition of the NET scaffold and the three recognized routes of NET formation. The composition question bears directly on the axis: NETs enriched in NE and cathepsin G will engage PAR2 differently from NETs dominated by histones and DNA, and differences in NET-associated protease content may help explain why PAR2 dependence measured in one cancer model does not reproduce in another (Sections 3.1 and 7).

**Figure 1:**
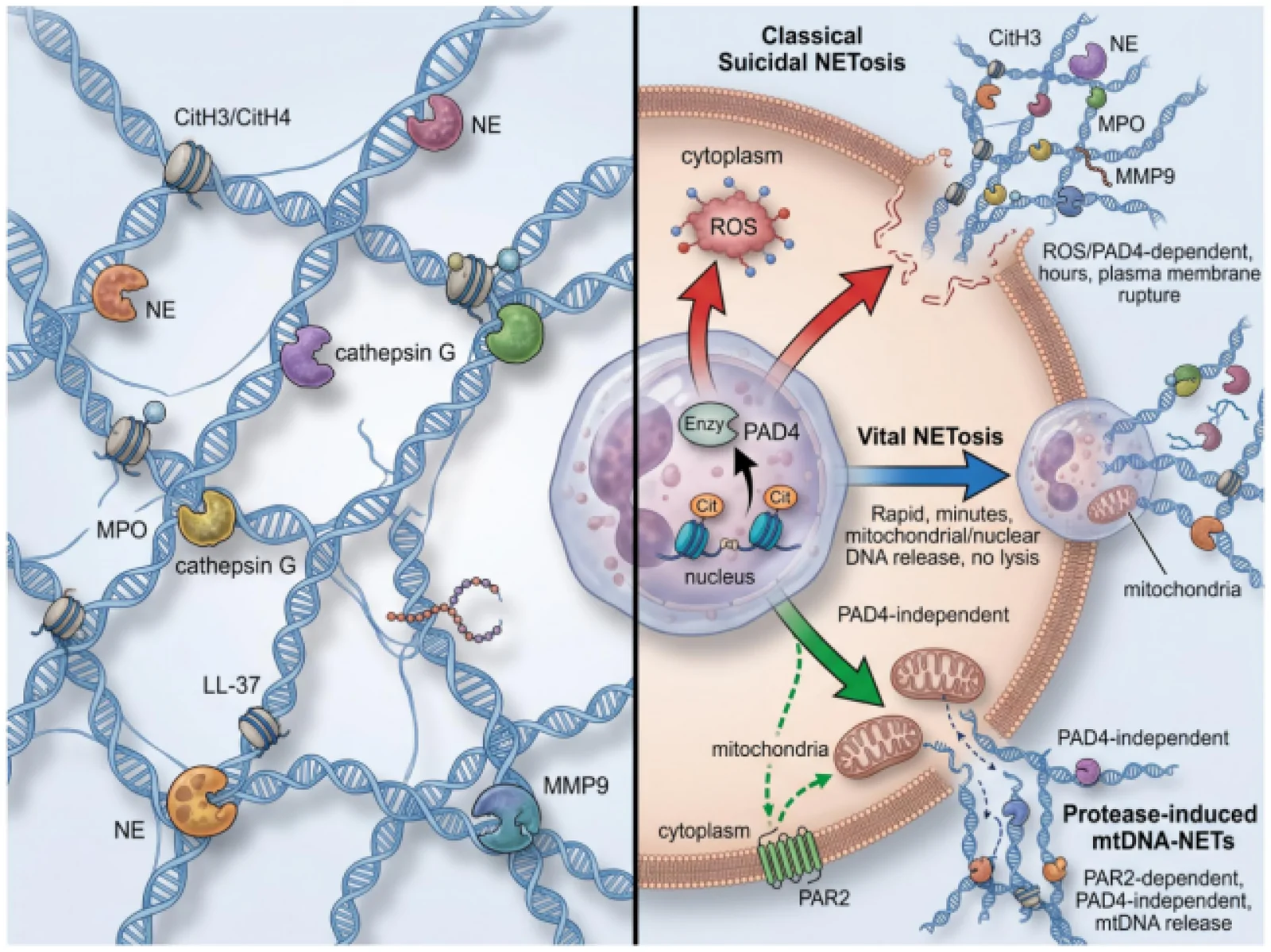
Illustration of NET structure and formation. Left panel: composition of the NET scaffold, comprising DNA fibers, citrullinated histones (CitH3/CitH4), NE, MPO, cathepsin G, LL-37, MMP9. Right panel: three routes of NET formation: classical suicidal NETosis (ROS- and PAD4-dependent, hours, plasma membrane rupture), vital NETosis (rapid, minutes, mitochondrial or nuclear DNA release without lysis), and protease-induced non-classical mtDNA-NETs (PAR2-dependent, PAD4-independent). Abbreviations are defined in the text.

NET release falls into two broad categories distinguished by stimulus and kinetics. Classical suicidal NETosis is a prolonged process lasting several hours that culminates in neutrophil death: ROS generation and PAD4-mediated histone citrullination decondense chromatin, after which the plasma membrane ruptures and nuclear chromatin is expelled. Vital NETosis, by contrast, occurs within minutes, preserves cell viability, and releases mitochondrial or nuclear DNA without membrane disruption; PMA, fungi, and immune complexes favor the suicidal route, whereas rapid bacterial stimulation and activated platelets favor the vital route (2, 3).

A third, non-classical subset has recently been characterized in the TME: NETs composed predominantly of mitochondrial DNA (mtDNA-NETs), generated independently of PAD4-mediated chromatin decondensation, induced by PAR2 signaling proteases, and engaging toll-like receptor 9 (TLR9) on tumor cells (10). Unlike classical NETs, mtDNA-NETs lack bactericidal activity yet carry a distinctive proteomic signature linking them to sterile inflammation and carcinogenesis (2, 10); their existence directly couples PAR2 signaling to NET production, closing a loop in which PAR2 both generates and responds to NET components.

These are biologically distinct directions of interaction that must not be conflated. Upstream, PAR2 signaling acts on neutrophils and shapes NET formation, as in the PAR2-dependent, PAD4-independent mtDNA-NETs described above (10). Downstream, NET components activate PAR2 on tumor and stromal cells to drive invasion (Sections 3-5). Evidence for PAR2-dependent NET formation does not by itself support the NET-PAR2 tumor-invasion axis, and in this review the two directions are always labeled explicitly.

The diversity of NET forms matters for cancer biology: the same molecular platform that defends against pathogens can, depending on context, drive immune modulation, metastasis, and therapy resistance. Understanding NET composition and classification is therefore a prerequisite for designing interventions that selectively disable pro-tumor functions while preserving host defense (3, 4).

### 2.2 Signaling pathways and regulatory factors in NET formation

NETosis is controlled by a layered regulatory network. PAD4-mediated histone citrullination is the rate-limiting step: it converts histone arginine residues to citrulline, relaxing chromatin so that DNA can be extruded. Genetic deletion or pharmacological inhibition of PAD4 abolishes NETosis, and in murine tumor models PAD4 deficiency or inhibition reduces metastatic nodule formation in the liver and lungs, reinforcing the contribution of PAD4-dependent NETs to tumor dissemination (15). These observations have made PAD4 the most intensively studied target for NET-directed therapy (Section 6.1).

Hypoxia is a major environmental driver. In neutrophils within the hypoxic tumor milieu, HIF-1α is stabilized and enhances PAD4 expression, while concurrently activating AKT-mTOR signaling that sustains neutrophil survival (29–31). Tumor cells further “pre-activate” circulating neutrophils through paracrine factors such as G-CSF and IL-8, lowering the threshold for NETosis (6), and Fusobacterium nucleatum, an oral commensal linked to colorectal cancer, promotes tumor progression by inducing NET formation via TLR4-ROS and NOD1/2 signaling (32).

Pattern recognition receptors and intracellular cascades add further control. TLR2 and TLR4 recognize PAMPs and DAMPs and engage MAPK pathways (p38, ERK1/2) and NADPH oxidase, providing the ROS burst required for NETosis (33); Mincle regulates NET formation through autophagy without affecting ROS (34); and extracellular purines modulate NET production, with adenosine acting on the A2A receptor inhibiting it (35), while Kindlin-3 negatively regulates NET release (36). Multiple inputs thus converge on ROS and PAD4, and the threshold for NET release is set by the integrated activity of cytokine, purinergic, and pattern-recognition signaling.

The layered design of this network has practical consequences. Because NETosis integrates so many inputs, a single upstream blockade rarely eliminates NET formation completely; the most effective interventions disable the final common pathway: PAD4-dependent citrullination or the NET scaffold itself (8, 15). NET abundance within a tumor is therefore a readout of the local inflammatory balance, so that circulating NET markers could serve as dynamic indicators of TME inflammation (Section 8.3) (6, 7).

### 2.3 Distribution characteristics of NETs in the tumor microenvironment

NETs are not uniformly distributed within tumors. They concentrate at the invasive margins, necrotic zones, and perivascular regions, the microanatomical niches where tumor cell dissemination and intravasation occur (7, 37). Quantification with DNA-binding dyes combined with MPO and CitH3 co-staining shows higher NET densities in aggressive malignancies such as triple-negative breast cancer (TNBC) than in indolent tumors (7, 37), and liquid biopsy studies detect circulating CitH3-DNA complexes that track tumor invasiveness and treatment response (6, 7).

Mechanistically, NETs at the tumor-stroma interface remodel the extracellular matrix and induce EMT, and they interact with stromal and immune cells to establish an immunosuppressive environment that impairs cytotoxic lymphocyte function (6, 7, 37). Preclinical interventions that dismantle NETs (DNase I) or prevent their formation (PAD4 blockade) reduce tumor growth and metastasis and partially restore anti-tumor immunity (8, 15); NET-targeted agents may be most effective when delivered to the invasive edge (Section 6.1) (38).

## 3 Structural features and signal transduction basis of PAR2

### 3.1 Molecular structure and activation mechanism of PAR2

PAR2 belongs to the class A G protein-coupled receptor family and differs from most GPCRs in how it is activated. Its extracellular N-terminus contains a cryptic tethered-ligand sequence (the hexapeptide SLIGKV in human PAR2); proteolytic cleavage exposes this sequence, which binds intramolecularly to the receptor extracellular loops and triggers G protein signaling (2, 39). This self-ligating mechanism explains why PAR2 activation is irreversible on the timescale of the encounter.

The tethered-ligand mechanism has an important pharmacological corollary: because the agonist is generated by proteolysis rather than by ligand binding, conventional competitive antagonists cannot prevent activation by occupying an orthosteric site. PAR2 antagonism therefore requires allosteric blockade or agents that prevent receptor coupling to G proteins, which is why the most effective PAR2 antagonists act as biased inverse agonists or antibodies that sterically occlude the tethered-ligand binding pocket (Section 6.2) (2, 11). Different proteases can also cleave the N-terminus at different sites, generating tethered ligands of differing potency and, in principle, differing signaling bias.

PAR2 has a broader protease activation profile than PAR1 or PAR4, being activated by trypsin, mast cell tryptase, NE, MMP2/MMP9, and the TF-FVIIa-FXa complex (9, 39). This promiscuity is directly relevant to NETs: NET-associated proteases and coagulation factors within tumors are well within PAR2’s activation spectrum, positioning the receptor as a molecular switch that translates extracellular proteolysis into intracellular signals. Regulatory checkpoints exist as well: RNF43 promotes PAR2 turnover in colon cancer (40), and neutrophil-derived serine proteases act on epithelial PAR2 signaling in a protective direction in the inflamed gut, where F2RL1/PAR2 deficiency aggravates colitis-associated carcinogenesis (41; Section 7.5).

Oxidative stress in the TME adds another layer of regulation by promoting PAR2 dimerization. Homodimers and PAR1-PAR2 heterodimers form under oxidative conditions, and heterodimerization with PAR1 can change ligand specificity and signaling bias. In particular, thrombin transactivation of the PAR1-PAR2 heterodimer elicits β-arrestin-mediated endosomal signaling that prolongs MAPK activation, a mode of sustained signaling linked to persistent pro-invasive phenotypes (42).

At the molecular level, PAR2 couples to Gαq/11, which activates phospholipase C and mobilizes intracellular calcium, and to Gα12/13, which engages RhoA and modulates cytoskeletal dynamics. β-arrestin recruitment following receptor phosphorylation desensitizes the receptor and scaffolds alternative signals such as ERK and JNK. The balance between G protein-dependent and β-arrestin-dependent signaling determines the biological output, which ranges from inflammation to tumor cell proliferation, migration, and angiogenesis (2, 42).

The pathological relevance of these features is well illustrated in cancer. In lung adenocarcinoma, PAR2 overexpression correlates with enhanced angiogenesis through EGFR activation (39); in hepatocellular carcinoma, PAR2 on hepatic stellate cells supports tumor growth and angiogenesis via MAPK (43); and hypomethylation of the PAR2 promoter increases receptor expression and is associated with poorer prognosis in lung adenocarcinoma (44). PAR2 function in tumors is thus shaped at three levels: genetic, biochemical, and cellular.

### 3.2 Downstream classical signaling pathways of PAR2

The signaling network engaged by PAR2 can be summarized by two canonical arms plus accessory scaffolds (**Figure 2**), presented here as receptor-level fundamentals; their functional consequences for invasion are developed in Chapter 4.

**Figure 2:**
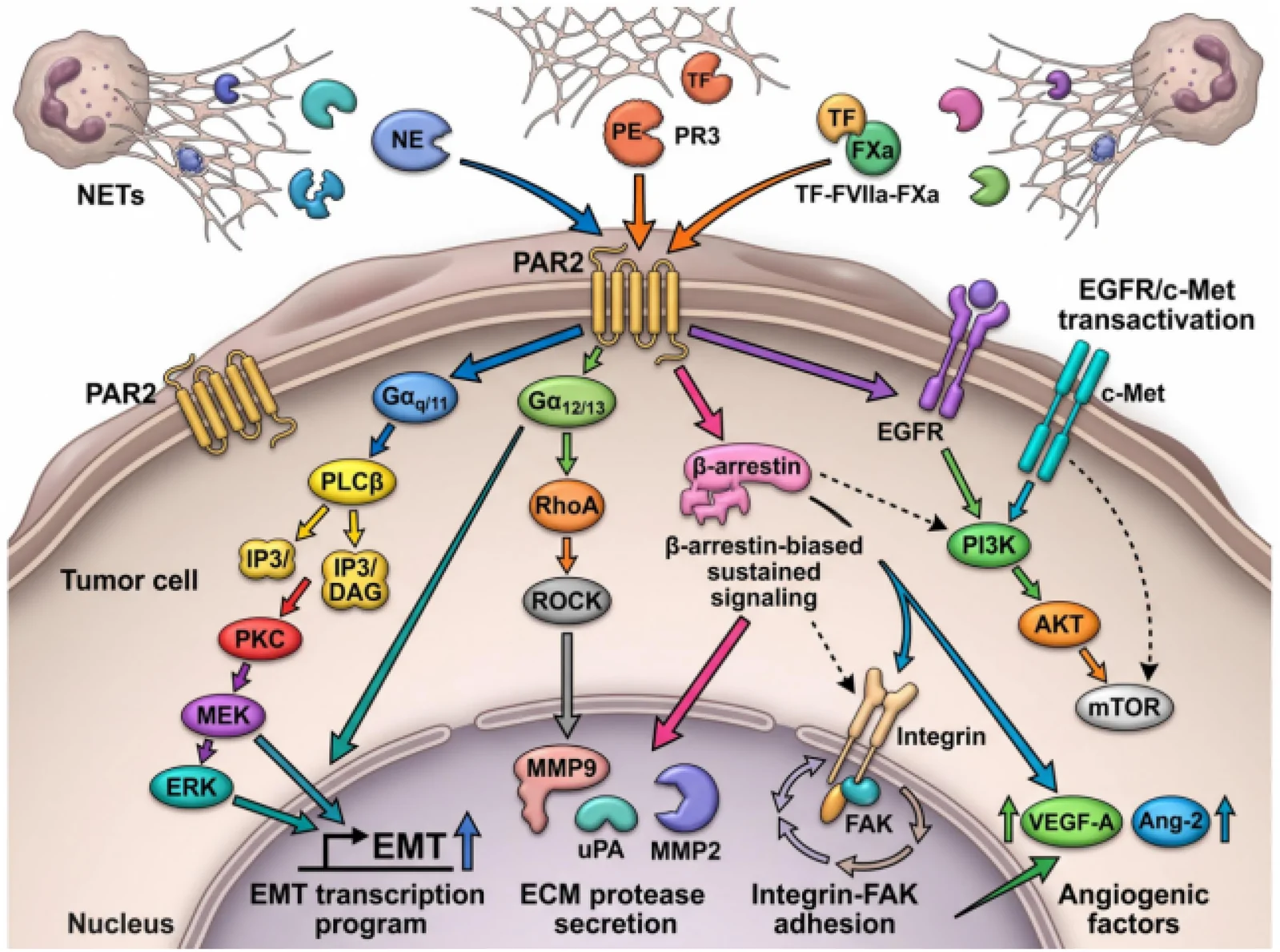
Overview of the NETs-PAR2 signaling network in tumor cells. NET-derived proteases (NE, PR3, TF-FVIIa-FXa) cleave and activate PAR2, engaging (i) Gαq/11-PLCβ-IP3/DAG-PKC-MEK-ERK, (ii) Gα12/13-RhoA-ROCK, (iii) β-arrestin-biased sustained signaling, and (iv) EGFR/c-Met transactivation feeding PI3K-AKT-mTOR. Downstream functional modules: EMT transcription programs, ECM protease secretion (MMP9/uPA/MMP2), integrin-FAK adhesion remodeling, and angiogenic factors (VEGF-A, Ang-2). Dotted lines indicate interactions supported by indirect or partial evidence. Responding cell types (tumor cells, endothelial cells, fibroblasts/stellate cells, macrophages) are indicated where known.

Gαq/11-PLCβ-PKC-MEK-ERK. PAR2 coupling to Gαq/11 stimulates phospholipase Cβ, which hydrolyzes PIP2 into IP3 and DAG; IP3 releases calcium from intracellular stores, while DAG activates protein kinase C (PKC). PKC in turn phosphorylates Raf-1 and initiates the MEK1/2-ERK1/2 cascade, culminating in the upregulation of genes such as MMP9 and urokinase-type plasminogen activator (uPA) that degrade the extracellular matrix and support cell migration (2). This arm is the best-characterized PAR2 pathway in tumor cells and is central to the EMT and protease programs discussed in Sections 4.1 and 4.2.

Gα12/13-RhoA-ROCK. PAR2 also couples to Gα12/13, activating RhoA and its effector Rho-associated kinase (ROCK). ROCK phosphorylates myosin light chain, promoting actin stress fiber formation and cellular contractility needed for movement through dense matrices; it also drives endocytosis and degradation of junctional proteins such as E-cadherin, a hallmark of EMT (2). This arm links PAR2 activation to the cytoskeletal remodeling that underlies amoeboid and collective modes of invasion.

Two accessory mechanisms extend these signals. β-Arrestin-1/2, recruited after receptor phosphorylation, scaffold Src and ERK cascade components, producing G protein-independent, sustained ERK activation that favors proliferation and apoptosis resistance; in the PAR1-PAR2 heterodimer, this prolonged phase is associated with endosomal signaling that supports chronic pro-invasive phenotypes (42). PAR2 additionally transactivates receptor tyrosine kinases such as EGFR and c-Met via MMP-dependent shedding of membrane-bound ligands such as HB-EGF, amplifying PI3K-AKT-mTOR signaling (39); the c-Met branch is particularly relevant because HGF, a c-Met ligand, is itself released by heparanase action downstream of PAR2 (Section 4.1), creating a self-amplifying loop (2).

### 3.3 PAR2 expression in different tissues and immune cells

PAR2 expression is regulated at the transcriptional level by transcription factors including Sp1 and AP-1, and promoter activity is enhanced by hypoxia, TGF-β, and TNF-α, conditions prevalent at the invasive front (2). NETs upregulate TF expression in breast cancer cells, and the pro-tumor effect is mediated through the TF/PAR2 axis (9). Transcriptional control of PAR2 is therefore coupled to the very microenvironmental signals that drive NET formation.

Beyond tumor cells, PAR2 is expressed on tumor-associated macrophages (TAMs) and myeloid-derived suppressor cells (MDSCs), where NETs engage PAR2 to promote secretion of immunosuppressive cytokines such as IL-10 and TGF-β (6). On endothelial cells, PAR2 mediates interactions between CTCs and the vascular wall, and circulating NETs can sequester tumor cells, promoting their adhesion and transendothelial migration (6). PAR2 on stromal fibroblasts and stellate cells contributes to the desmoplastic reaction in several tumor types (Section 7.2), indicating that the receptor’s pro-tumor effects are distributed across the cellular ecosystem of the tumor.

The net picture is a receptor whose expression is tuned by the same microenvironmental stressors that drive NET formation. Invasive tumor cells therefore become maximally sensitive to NET-derived proteases precisely where NETs accumulate, while PAR2 on myeloid and endothelial cells extends the axis into immune suppression and vascular dissemination. This distributed expression pattern is both the strength and the challenge of the axis as a therapeutic target.

Two additional expression sites define the safety envelope of the axis. PAR2 on peripheral neurons mediates pain and pruritus; chronic PAR2 blockade would therefore be expected to carry analgesic effects, which could be either a benefit or a confounder in cancer patients (45, 46). PAR2 on platelets contributes to platelet activation and thrombosis, relevant because NETs are potent prothrombotic scaffolds and cancer patients are at elevated thrombotic risk (6, 9). Any therapeutic strategy must weigh antitumor efficacy against these physiological roles (Section 8.4).

## 4 Direct evidence of NETs-PAR2 signaling axis driving tumor invasion

### 4.1 Key findings in *in vitro* co-culture and 3D invasion models

Co-culture experiments in which neutrophils are stimulated to form NETs consistently show increased invasive capacity of tumor cells in Transwell and Matrigel assays. This response depends critically on PAR2: pharmacological or genetic disruption of the TF/PAR2 pathway attenuates NET-induced pro-tumor gene expression and invasion in human breast cancer cells (9). The evidence, however, comes largely from TF/PAR2 pathway manipulations rather than from systematic PAR2−/− or GB88 loss-of-function experiments, so the full extent to which NET-induced invasion depends on PAR2 alone remains to be established (9, 10).

Three-dimensional models add spatial resolution. In tumor spheroids and organoids, NETs promote directional budding of tumor cells into the surrounding matrix and upregulate integrins αvβ3 and α5β1 that bind fibronectin and vitronectin (6). Microfluidic tumor-immune microenvironment (TIME)-on-chip platforms show collective, NETs-mediated invasion into a three-dimensional neutrophil-containing matrix, with extracellular DNA and NE contributing chemotactic cues (47). In the TIME-on-chip system, drug-induced inhibition of NET formation reverses this collective invasion (47); head-to-head dissection of whether PAR2 engagement or NE enzymatic activity is the dominant sensing mechanism within such 3D systems has not been performed, and the question remains open. The strongest available evidence comes from separate models, in which the anti-PAR2 blocking antibody mAb26685 reduces primary-tumor invasion depth and metastatic burden in xenografts (9).

A related observation comes from cholangiocarcinoma, where NETs act as a physical scaffold that promotes cancer progression and modulates angiogenesis through the ITGAV/NF-κB axis, illustrating how NET architecture itself, independent of soluble proteases, can organize tumor-stroma communication (27). This scaffold function, in which NETs act as a three-dimensional platform concentrating proteases, growth factors, and adhesion ligands, may explain why complete inhibition of NET-driven invasion requires dismantling the NET structure (DNase I) in addition to blocking PAR2.

Two caveats accompany the interpretation of these assays. First, most co-culture experiments use NETs generated ex vivo by PMA or bacterial stimulation, whose density and composition differ from intratumoral NETs; the quantitative PAR2 dependence measured *in vitro* may therefore not translate directly to the clinic (Section 8). Second, Transwell and Matrigel assays measure population-level migration into a homogeneous matrix, whereas native invasion involves gradients of chemokines, matrix stiffness, and immune cells; the concordance of results across Transwell, spheroid, and microfluidic platforms nevertheless strengthens the conclusion that PAR2 is a genuine mediator of NET-induced invasion (6, 47).

### 4.2 Validation and quantitative analysis in *in vivo* animal models

*In vivo* models link NETs to metastasis at the organismal level (48). In tail vein metastasis models, systemic induction of NETs before tumor cell injection increases the number of lung metastatic nodules; conversely, preventing NET formation with the PAD4 inhibitor Cl-amidine or genetic PAD4 knockout, or digesting NETs with DNase I, significantly reduces lung metastatic burden, with decreased ERK phosphorylation in lung tissue (8, 15). Park et al. demonstrated that metastatic breast cancer cells induce NET formation in the lungs and that DNase I-coated nanoparticles markedly reduce lung metastases (8). The magnitude of the effect varies between models, and the precise contribution of PAR2 downstream of these manipulations has not been isolated in most studies, a gap that matters because PAD4 and DNase I remove NETs entirely, whereas a PAR2-specific intervention would test the receptor’s specific role.

Orthotopic models provide complementary evidence for local invasion. In mammary fat pad models, intratumoral administration of DNase I reduces the depth of tumor invasion, with decreased MMP9 activity and vimentin expression at the invasive front, together with molecular features of EMT associated with suppression of the PAR2-ERK1/2 cascade (8, 9). The convergence of *in vitro*, 3D, and orthotopic studies on the same molecular readouts (MMP9, vimentin, ERK phosphorylation) strengthens the case that the NETs-PAR2-ERK axis is a conserved driver of the invasive phenotype.

Host-stroma PAR2 appears to be at least as important as tumor-cell PAR2. In syngeneic models (MC38 colon carcinoma, Lewis lung carcinoma) implanted in PAR2-deficient mice, tumors exhibit more regular, well-demarcated boundaries with reduced infiltration into adjacent muscle, suggesting that stromal PAR2 cooperates with tumor-cell signaling to create a permissive microenvironment for NET-driven invasion (9). If host PAR2 is the dominant driver, tumor-cell-autonomous studies will underestimate the axis’s contribution, and PAR2-targeted therapies would need to reach stromal as well as tumor compartments.

### 4.3 Correlation and prognostic value in clinical samples

Most clinical evidence for the NETs-PAR2 axis in patients measures NET burden rather than PAR2 engagement directly. In metastatic breast cancer, circulating NET markers are elevated and independently associated with poor survival and a hypercoagulable state (49); in pancreatic ductal adenocarcinoma, blood-borne NETs discriminate patients from controls and hold promise as diagnostic biomarkers (50); and pancreatectomy induces a systemic increase in cancer-promoting NETs, providing a plausible mechanistic link between perioperative inflammation and early postoperative recurrence (51). These studies establish that NETs form and accumulate in cancer patients at clinically meaningful time points, but they stop short of showing where, and through which receptor, the trapped proteases act.

Transcriptomic approaches have begun to connect NET biology to outcome at the tissue level. NET-related gene signatures carry prognostic information in colorectal cancer and its liver metastases (25), and the PAR-family gene F2RL2 emerges in a NET-related prognostic signature in breast cancer cohorts (18); because F2RL2 encodes PAR3 rather than PAR2 (F2RL1), this is prognostic evidence for NET-related biology rather than for the NET-PAR2 axis itself (Section 7.1). What is still missing is a validated tissue assay that documents PAR2 engagement by NET-derived proteases in human tumors: proximity ligation detection of protease-PAR2 complexes at tumor cell membranes, or paired imaging of NET deposition with downstream ERK activation, would close this loop directly and represents one of the most urgent unmet needs in the field (Section 8.3).

A note on interpretation is warranted here. Correlation between NET/PAR2 readouts and tumor aggressiveness does not by itself establish that the axis drives invasion; the association could reflect reverse causality (aggressive tumors recruit more neutrophils) or confounding by inflammation. The strongest causal evidence remains experimental, namely PAR2 loss-of-function and NET depletion studies in model systems (Sections 3.1 and 3.2), and the clinical data are best read as confirming that the molecular events demonstrated in the laboratory actually occur in patients (6, 7).

## 5 Molecular mechanisms of NETs-PAR2 signaling axis promoting tumor invasion

Once NET-derived proteases activate PAR2 on tumor and stromal cells, the signal is distributed across four partially overlapping mechanistic modules: extracellular matrix remodeling by proteases, epithelial-mesenchymal transition, blood and lymphatic vessel neoformation, and construction of an immunosuppressive microenvironment. These modules, summarized in **Figure 3**, should be read as concurrent arms of a single program: degraded matrix releases sequestered growth factors that potentiate EMT, EMT-derived VEGF reinforces angiogenesis, and the resulting vascular and immune changes further favor NET formation. Because the biological outcome of PAR2 activation differs substantially between cell types, we specify the responding population (tumor cell, endothelial cell, fibroblast or stellate cell, macrophage) wherever the primary studies allow.

**Figure 3:**
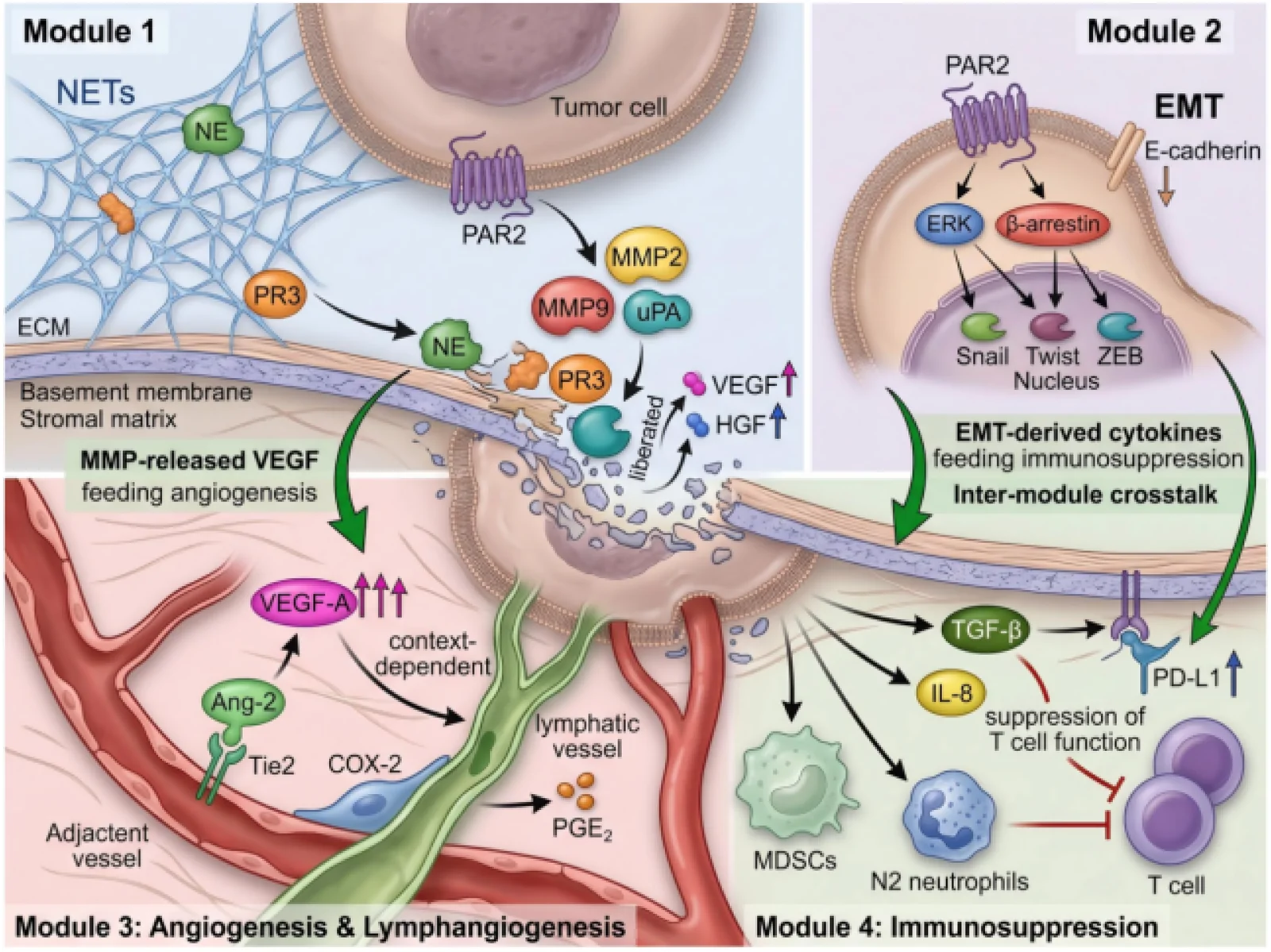
Overview of the four mechanistic modules through which NETs-PAR2 signaling promotes tumor invasion. Module 1, ECM remodeling: NET-shuttled NE/PR3 and PAR2-induced MMP2/MMP9/uPA degrade basement membrane and stromal matrix, liberating matrix-bound VEGF and HGF. Module 2, EMT: ERK- and β-arrestin-dependent activation of EMT transcription factors (Snail, Twist, ZEB) with loss of E-cadherin. Module 3, angiogenesis and lymphangiogenesis: VEGF-A upregulation, Ang-2/Tie2 signaling, COX-2-derived PGE2, and context-dependent lymphatic effects. Module 4, immunosuppression: TGF-β and IL-8 secretion, MDSC recruitment, N2 neutrophil polarization, T-cell suppression, and PD-L1 induction. Inter-module arrows indicate documented crosstalk (e.g., MMP-released VEGF feeding angiogenesis; EMT-derived cytokines feeding immunosuppression).

### 5.1 Protease profile remodeling and extracellular matrix degradation

NETs deliver NE and proteinase 3 to the tumor-stroma interface, where they activate PAR2 on cancer cells and trigger the *de novo* synthesis and secretion of matrix metalloproteinases, especially MMP9 and MMP2. These tumor-derived MMPs join the MMP9 pool already anchored on NET fibers, forming a concentrated proteolytic depot that degrades basement membrane and interstitial collagen and opens paths for invading cells (2, 6). The tumor-stroma interface thus accumulates a high local concentration of both NET-anchored and tumor-secreted enzymes, producing a microenvironment structurally permissive for invasion.

PAR2 activation also influences the membrane-tethered protease machinery. PAR2 stimulation upregulates membrane-type 1 matrix metalloproteinase (MT1-MMP/MMP14), which both degrades type I collagen and laminin and activates pro-MMP2; in breast cancer cells, coagulation protease-driven PAR2 signaling promotes MMP-2-mediated invasion through autocrine signaling (52). In pancreatic cancer cells, PAR2 agonists increase proliferation and invasion in a manner associated with MMP2 expression (53). These findings support a model in which PAR2 couples NET-derived proteases to an amplified MMP cascade, with MT1-MMP at the apex amplifying the proteolytic output of the entire system.

PAR2 signaling additionally induces heparanase, an endoglycosidase that cleaves heparan sulfate proteoglycans and releases sequestered growth factors such as FGF and HGF (2). Heparan sulfate chains in the basement membrane act as a reservoir for growth factors; their cleavage by heparanase liberates these ligands and simultaneously fragments the matrix. HGF has been identified as a NETs-related biomarker in multiple myeloma, emphasizing the clinical relevance of growth-factor liberation downstream of NETs (54). Heparanase’s coupling to the c-Met/HGF loop (Section 2.2) makes it a plausible amplifier of the entire axis, and existing heparanase inhibitors merit evaluation in NETs-PAR2-driven models (2).

Two caveats temper this picture. First, the NET-induced EMT contribution of the TF/PAR2 axis in breast cancer is supported mainly by gene-expression studies, with limited direct invasion measurements (9). Second, NET-derived NE can inactivate canonical PAR2 signaling and thereby downregulate CD24, promoting macrophage phagocytosis of colorectal cancer cells (11; Section 7.5). The proteolytic output of the axis is therefore context-dependent: the same proteases that drive matrix degradation and invasion in one setting can, by cleaving PAR2 at non-canonical sites, switch the receptor’s signaling and downstream effects in another.

### 5.2 Induction and regulation of epithelial-mesenchymal transition

EMT, the loss of epithelial markers such as E-cadherin together with the gain of mesenchymal markers such as N-cadherin, fibronectin, and vimentin, is a central mechanism through which NETs increase tumor cell motility and invasiveness. Stimulation of tumor cells with NETs produces a time-dependent EMT signature at the protein level, and PAR2 signaling is proposed as the principal conduit for this effect (6). EMT spans a spectrum of intermediate states, and NET-driven signals appear to push tumor cells toward a full mesenchymal phenotype, with consequences for both motility and therapy resistance.

Mechanistically, PAR2-dependent activation of the PKC-MEK-ERK pathway is thought to drive the EMT transcriptional program; ERK-mediated phosphorylation of EMT transcription factors such as Snail promotes their nuclear accumulation and repression of E-cadherin expression, although direct evidence for specific phosphorylation sites is lacking and the claim should be read as a working model. In hepatocellular carcinoma, PAR2 promotes proliferation, EMT, and metastasis (55). In NSCLC, PAR2 blockade reverses osimertinib resistance at least in part by attenuating ERK-mediated EMT (13), and similar ERK-EMT coupling is observed in pancreatic cancer cells treated with PAR2 agonists (53). The repeated co-occurrence of PAR2 activation, ERK signaling, and EMT across tumor types supports the generalizability of this axis.

A distinctive feature of the PAR2-EMT axis is its cooperation with TGF-β. PAR2 activation enhances expression of the TGF-β receptor ALK5 and potentiates Smad2/3 C-terminal phosphorylation; combined PAR2 and TGF-β stimulation produces stronger Smad transcriptional activity than either alone, driven by ERK-mediated phosphorylation of the Smad linker region (6, 9). In breast and pancreatic cancer models, combined PAR2 and TGF-β input upregulates EMT transcription factors such as Snail and ZEB1 more completely than either signal alone (6, 9). The ERK-Smad linker module is thus a convergence point integrating inflammatory (PAR2) and developmental (TGF-β) cues into a single transcriptional program, which is clinically significant because TGF-β is abundant in most tumor microenvironments. In most models this cooperation was established with synthetic PAR2 agonists or coagulation proteases rather than with NET-derived proteases directly, so the NET contribution remains inferential (emerging).

### 5.3 Angiogenesis and lymphangiogenesis

PAR2 activation promotes tumor angiogenesis through both autocrine and paracrine routes. PAR2-stimulated tumor cells secrete VEGF-A, which acts on VEGFR-2 on adjacent endothelial cells to drive proliferation, lumen formation, and sprouting, increasing microvascular density (2, 39). In lung adenocarcinoma, the PAR2-EGFR connection is particularly well documented: PAR2 overexpression correlates with enhanced tumor-associated angiogenesis through EGFR pathway activation (39), and PAR2 on hepatic stellate cells similarly drives pro-angiogenic factor secretion in hepatocellular carcinoma (43).

NETs further potentiate vascular remodeling by inducing angiopoietin-2 (Ang-2) in tumor-associated endothelial cells through PAR2. Ang-2 destabilizes vessels via the Tie2 receptor, increasing permeability and facilitating tumor cell extravasation. In an experimental model of retinopathy of prematurity, PAR2 activation increased TNF-α-dependent Ang-2 expression and Tie2-dependent angiogenesis, establishing a PAR2-Ang-2-Tie2 pathway likely conserved in tumors (56). The NETs-PAR2-Ang-2 axis thus feeds into the leaky, tortuous vessels characteristic of aggressive tumors (27, 56).

The role of the axis in lymphangiogenesis is more controversial. One influential study reported that PAR2 activation suppresses lymphangiogenesis and subsequent lymph node metastasis in a murine pancreatic cancer model (57), whereas NETs have been linked to lymph node metastasis in cervical cancer, where S100A7 orchestrates neutrophil chemotaxis and NET formation (28). The effect of PAR2 on lymphatic vessels is therefore context-dependent: the blood-vascular effects of the axis (VEGF-A, Ang-2) are well supported, whereas its lymphatic effects require further study and may even be inhibitory in some models (28, 57).

### 5.4 Inflammation and immunosuppressive microenvironment formation

NETs and PAR2 contribute to an immunosuppressive TME through several reinforcing mechanisms. PAR2 activation in tumor cells and TAMs promotes secretion of chemokines (CCL2, CCL5, CXCL12) that recruit CCR2/CXCR4-expressing myeloid-derived suppressor cells and regulatory T cells into the tumor (6). PAR2 signaling also drives TAM polarization toward an M2 phenotype; M2 macrophages in turn secrete IL-8 and TGF-β that stimulate further NETosis, creating a self-perpetuating “NETs-PAR2-M2 macrophage-NETs” loop (emerging evidence; direct demonstration of NET-derived proteases activating PAR2 on TAMs is still lacking) (6, 58).

The impact on cytotoxic T cells is direct, but it is attributable to NETs themselves rather than to PAR2 signaling. In human tumor xenograft models, NETs substantially reduce CD8+ T cell infiltration and upregulate exhaustion markers including PD-1 and LAG-3 on residual tumor-infiltrating T cells (59). This distinction matters: the study demonstrates a NET effect on T cell exhaustion, not NET-PAR2 signaling, and no published experiment has yet shown that NET engagement of PAR2 on immune populations is required for T cell suppression. The upregulation of multiple inhibitory receptors suggests NET-driven suppression produces a relatively advanced exhaustion state that may be resistant to single-agent checkpoint blockade, an argument for combining NET-directed therapies with immunotherapy (Section 6.3).

Three non-mutually exclusive mechanisms can account for the T cell effects. NETs physically entrap T cells in their DNA-protein mesh, limiting their mobility and access to antigen-presenting cells; they present self-DNA and oxidized mitochondrial DNA that can engage TLR9 on T cells and drive tolerogenic signaling; and they remodel the chemokine milieu, sequestering CXCL10 and other T cell-attracting chemokines (6, 59). Because several of these mechanisms are PAR2-independent, effective reversal of NET-driven T cell suppression may require both NET clearance (DNase I) and PAR2-directed intervention (Section 6.3) (6); attribution of the exhaustion phenotype specifically to NET-PAR2 signaling must await direct testing.

The immunomodulatory role of PAR2 is, however, not uniformly immunosuppressive. PAR2 deficiency in a colorectal cancer model impairs antitumor immunity and attenuates anti-PD-1 efficacy, indicating that PAR2 signaling can support productive antitumor responses in certain contexts (14; see Section 7.5). The most productive framework is to move beyond “is PAR2 pro- or anti-tumorigenic” and instead ask which PAR2-expressing cell type, in which context, produces which outcome (Section 6.3).

## 6 Crosstalk between the NETs-PAR2 signaling axis and other signaling pathways

### 6.1 Crosstalk with the Wnt/β-catenin pathway

The NETs-PAR2 axis intersects with Wnt/β-catenin signaling at several points, converging on shared transcriptional outputs that coordinate proliferation with invasion. PAR2 activation has been reported to stabilize β-catenin and promote its nuclear accumulation, where it forms a transcriptionally active complex with TCF/LEF transcription factors (60, 61). In colorectal cancer, PAR2 signaling supports cancer cell self-renewal and metastasis through β-catenin and periostin (60). A complementary route runs through the PAR2-COX-2-β-catenin cascade: PAR2 induces cyclooxygenase-2 expression via β-catenin and CREB, and the prostaglandins produced can further stabilize β-catenin, creating a positive loop that sustains Wnt output (61). Neither route requires genetic mutation of the canonical Wnt machinery; both depend on the inflammatory and proteolytic milieu that NETs create.

Downstream of β-catenin stabilization, target genes such as cyclin D1 and c-Myc drive G1/S progression, operating in parallel with the EMT transcription factors induced by PAR2-ERK signaling, so the two arms jointly coordinate cell-cycle entry with the loss of epithelial identity (60, 61). In colorectal cancer, the PAR2-β-catenin-periostin program links receptor activation to a stem-like, metastasis-prone transcriptional state (60). This convergence is driven by inflammation, not mutation: PAR2 activation stabilizes β-catenin in a ligand-dependent manner, so that in tumors without APC or CTNNB1 mutations, NET-derived proteases can transiently activate Wnt output. PAR2-driven β-catenin/TCF and CREB activation is functionally required for COX-2 induction in epithelial cells (61), although whether NET-derived proteases engage this specific Wnt branch has not been tested directly; an earlier proposal that protein kinase C δ directly phosphorylates LRP6 lacks direct experimental evidence and should be treated as speculative. Combining PAR2 antagonists with Wnt-pathway inhibitors may suppress the invasive phenotype more effectively than either agent alone, although this has not yet been tested *in vivo*.

### 6.2 Coupling with integrin/FAK signaling axis

PAR2 activation shapes focal adhesion dynamics through the integrin/FAK axis, controlling the motile branch of the NET-driven invasive program relatively independently of the protease-secretion branch. A critical early event is autophosphorylation of FAK at Tyr397, creating a high-affinity docking site for Src family kinases; Src recruitment leads to phosphorylation of paxillin and vinculin, accelerating focal adhesion turnover, essential for directional, persistent migration (2, 6). PAR2 stimulation also upregulates integrins αvβ3 and α5β1 (receptors for vitronectin and fibronectin), so that cells preferentially engage the provisional matrix proteins abundant at the NET-rich invasive front (6).

FAK inhibition with the selective small molecule VS-4718 reversibly abolishes the enhanced directional migration of tumor cells induced by NETs, while leaving PAR2-mediated MMP9 upregulation intact, showing that the integrin-FAK branch governs adhesion turnover and motility, whereas the protease branch controls matrix remodeling (6, 47). This separation has therapeutic consequences: agents targeting integrin-FAK could selectively impair NET-induced motility without broadly suppressing PAR2-dependent proteolysis, and vice versa. In pancreatic cancer, PAR2-mediated FAK activation strengthens integrin-dependent adhesion to the desmoplastic matrix and supports collective invasion (6, 19); the FAK-integrin arm thus drives single-cell movement in loose matrices but coordinated collective invasion in dense fibrotic tissue.

### 6.3 Mutual reinforcement with hypoxia/HIF-1α pathway

Hypoxia and the NETs-PAR2 axis reinforce each other through a positive feedback loop operating at the level of ligand production, receptor expression, and downstream effector signaling. Hypoxic conditions stabilize HIF-1α both in tumor cells and in infiltrating neutrophils, where the transcription factor upregulates PAD4 and favors NET formation (30, 31, 62); simultaneously, hypoxia transcriptionally upregulates PAR2 on tumor cells (2, 30). Hypoxia thus raises both the supply of the ligand (NETs) and the responsiveness of the receptor (PAR2), the precise arrangement required for a self-amplifying circuit.

The loop continues downstream. Both NETs and PAR2 signaling engage ERK and PI3K, either of which can enhance HIF-1α translation and stability in tumor cells, potentiating hypoxia-adaptive programs such as glycolysis, invasion, and angiogenesis (30, 63). Direct demonstration that NETs engage PAR2 on tumor cells to stabilize HIF-1α is, however, still lacking. Several experimental observations anchor this cycle in specific tissues: in gastric cancer, hypoxia induces NET formation via the HMGB1/TLR4/p38 MAPK pathway (30); HIF-1α-dependent glycolysis sustains the metabolic demands of NETosis (31, 62); in breast cancer models, HIF-1α-high NETs facilitate circulating tumor cell colonization and metastasis, and HIF-1α targeting suppresses metastatic progression (63); and NET-mediated HIF-1α upregulation in endothelial cells promotes corneal and choroidal neovascularization, indicating that the hypoxia-NETs-angiogenesis link is a general feature of NET biology rather than a cancer-specific artifact (64, 65).

The practical implication is that PAD4 inhibitors, PAR2 antagonists, and HIF-1α inhibitors each interrupt this loop at a different node. PAD4 blockade cuts NET production at its source; PAR2 antagonism dampens receptor-driven stabilization of HIF-1α; and HIF-1α inhibition removes the transcriptional driver of both adaptive metabolism and further NETosis. Preclinical data indicate that single-node interruption slows but may not fully terminate the cycle, arguing for combination regimens (30, 63). Because chronic hypoxia is a hallmark of aggressive solid tumors, this loop is likely to be most therapeutically relevant in exactly the tumors where NETs and PAR2 signaling already correlate with poor outcome.

The loop also connects hypoxia to immunotherapy resistance: HIF-1α-driven NETosis and PAR2 signaling promote the immunosuppressive features described in Section 4.4, and the hypoxic regions where NETs accumulate are precisely where effector T cells are least effective. Vascular normalization strategies that relieve tumor hypoxia could therefore dampen NET formation, PAR2 signaling, and immunosuppression simultaneously (12, 30).

## 7 Intervention strategies and therapeutic prospects targeting the NETs-PAR2 signaling axis

### 7.1 Inhibitors targeting NET formation

Because NET formation precedes PAR2 engagement, inhibiting NETosis blocks the axis at its earliest step and has become the most mature therapeutic strategy. The leading class targets PAD4, whose citrullination of histones is rate-limiting for chromatin decondensation. Cl-amidine blocks the formation of invasion-promoting NETs in metastasis models (8), and in breast cancer models PAD4 inhibition curtails NET-driven progression and metastasis, with genetic PAD4 deficiency phenocopying the pharmacological effect (15). The optimized pan-PAD inhibitor BB-Cl-amidine also reduces liver metastatic burden in experimental colorectal cancer models (66); in that study, however, the predominant PAD4 source is the cancer cell itself, which citrullinates extracellular matrix collagen to promote adhesion and a more epithelial phenotype rather than driving NET formation, so the effect validates PAD4 dependence rather than NET dependence. Note that these are PAD4- or NET-directed, not PAR2-directed, interventions: they validate the upstream dependence of progression but not the specific role of PAR2. BB-Cl-amidine has advanced into clinical development, and its tolerability profile will be a key test of whether chronic PAD4 blockade is feasible; on-target toxicity remains a risk because PAD4 may have physiological roles beyond NETosis.

DNase I offers a complementary strategy by dismantling NETs that have already formed, and has been studied most extensively in pancreatic and breast cancer models, where it synergizes with chemotherapy. In pancreatic ductal adenocarcinoma, gemcitabine itself induces NET formation through the IL-8-CXCR1/2 axis, and this chemotherapy-driven NETosis contributes to chemoresistance; inhibiting CXCR1/2 suppresses NET formation, restores gemcitabine sensitivity, and reduces tumor growth *in vivo* (23). DNase I treatment in breast cancer models reduces NET-induced metastasis, and DNase I-coated nanoparticles markedly reduce lung metastases in a tail-vein model (8, 67). These results have moved DNase I into early-phase clinical trials as a chemotherapy adjunct; a key limitation is pharmacokinetic: systemic DNase I must reach the NET-rich invasive front, motivating local delivery approaches (Section 8.4).

Upstream inhibitors target the signaling that generates NETs; NET-directed antitumor strategies have been comprehensively reviewed elsewhere (68). NADPH oxidase blockers such as diphenyleneiodonium (DPI) eliminate the ROS burst required for classical suicidal NETosis, and neutrophil elastase inhibitors such as sivelestat reduce the proteolytic activity of NETs; in colorectal cancer, DPI combined with integrin α5β1 antagonists suppresses NET-mediated metastasis (69). A growing pharmacopeia of natural products and repurposed drugs expands the options: kaempferol inhibits NET formation via the ROS-PAD4 pathway and reduces breast cancer metastasis (70); triptolide suppresses NET production independently of ROS (71); icaritin inhibits PADI2-mediated citrullination and NET formation in urothelial cancer, with data suggesting synergy with immune checkpoint inhibitors (72); and selinexor markedly suppresses NET release *in vitro* (73). Biomaterial-based delivery adds a further dimension: an injectable fibrin-alginate hydrogel co-delivering DNase I nanoparticles and propranolol disrupts NETs and antagonizes cancer-associated neurotransmitter signaling, suppressing relapse after surgical resection (74). Surgery itself is a potent trigger of NET formation, and local delivery saturating the wound bed with NET-degrading activity during the immediate postoperative window may be more effective than systemic therapy timed to later stages (8, 74).

### 7.2 Development of PAR2 antagonists and antibody drugs

Small-molecule PAR2 antagonists have reached an advanced stage of preclinical validation. GB88, an orally bioavailable peptidomimetic, and its analogs show dose-dependent inhibition of NET-induced tumor cell invasion and migration (11). Because PAR2 uses a tethered-ligand activation mechanism, conventional competitive antagonists cannot prevent activation; the most effective agents act as biased inverse agonists or antibodies that sterically occlude the tethered-ligand binding pocket (6, 11). Monoclonal antibodies targeting the PAR2 extracellular domain, such as mAb26685, block access of the tethered ligand to its binding pocket; in xenograft models, monotherapy reduced the depth of primary tumor invasion and the incidence of lung and bone metastases (9). Pharmacological or genetic inhibition of the TF/PAR2 axis likewise attenuates NET-induced pro-tumor gene expression in human breast cancer cells (9).

The past decade has produced a small but pharmacologically diverse toolbox of PAR2 antagonists. GB88, the first orally active small-molecule antagonist, behaves as a pathway-selective biased ligand: it suppresses Gq-dependent calcium signaling and inflammation *in vivo* while leaving β-arrestin recruitment largely intact (11, 75, 76). This bias cuts both ways for cancer: β-arrestin-mediated signaling contributes to PAR2-driven migration and EMT (Section 2.2), so an antagonist that spares this arm may underperform in anti-invasive assays. I-191, a subsequent-generation antagonist, blocks the calcium, ERK, RhoA, and β-arrestin arms simultaneously and inhibits PAR2-driven migration and invasion of human colon and ovarian cancer cells (77). AZ8838 occupies an intracellular allosteric pocket revealed by the PAR2 crystal structure (78); because it does not compete at the extracellular cleavage site, it inhibits signaling regardless of which protease performs the activating cleavage, of particular value in the NET setting, where NE, PR3, cathepsin G, and coagulation proteases act in parallel.

Emerging modalities aim for more complete receptor abrogation. Nanobody-drug conjugates combine the tissue penetration of single-domain antibodies with cytotoxic payloads and are being explored for PAR2-targeted delivery; PROTACs (proteolysis-targeting chimeras) recruit an E3 ligase to PAR2, converting transient blockade into sustained removal of the signaling pool. Both strategies remain at the proof-of-concept stage, with on-target toxicity and the consequences of eliminating (rather than merely blocking) a receptor with physiological roles in pain sensing, barrier function, and host defense as the main unresolved questions (2). The risk-benefit calculus of chronic PAR2 blockade has not been examined clinically, and this gap will define how quickly PAR2-directed drugs reach the clinic: despite two decades of medicinal chemistry, no PAR2 antagonist has progressed to late-phase clinical trials, owing to assay-dependent pharmacology, species differences in tethered-ligand recognition, and the absence of a validated pharmacodynamic biomarker (79). A candidate PAR2 antagonist should therefore be paired with a tissue pharmacodynamic assay (e.g., inhibition of NET-induced ERK phosphorylation in tumor biopsies) and a patient-selection strategy based on NET burden and receptor-pathway activity.

### 7.3 Exploration of combined immunotherapy strategies

The interaction between NETs, PAR2, and immune checkpoints is emerging as a determinant of immunotherapy response. In murine models resistant to anti-PD-1 therapy, NET burden is associated with reduced CD8+ T cell infiltration and increased exhaustion marker expression, and DNase I-mediated NET clearance enhances the efficacy of anti-PD-1 antibodies (12). In hepatocellular carcinoma, NETs promote immune escape by upregulating CD73 through Notch2, a pathway that dampens T cell responses independently of PD-L1 (12). The two examples illustrate distinct mechanisms by which NETs blunt immunotherapy: a quantitative effect on CD8+ T cell recruitment, and a metabolic-immune checkpoint effect.

PAR2 blockade may directly modulate the PD-1/PD-L1 axis. In NSCLC cells, PAR2 blockade reverses osimertinib resistance by attenuating ERK-mediated EMT and reducing PD-L1 expression (13); the relative contribution of STAT3 and NF-κB, two transcriptional regulators of PD-L1 downstream of PAR2, remains incompletely defined. PAR2 signaling is not uniformly immunosuppressive: in a colorectal cancer model, PAR2 deficiency impaired antitumor immunity and attenuated anti-PD-1 efficacy (14). The outcome of PAR2-targeted combination therapy therefore likely depends on tumor type, the dominant PAR2-expressing cell population, and the pre-existing immune contexture.

NET clearance may also improve the trafficking of adoptively transferred cells. DNase I-mediated NET digestion restores CD8+ T cell infiltration in preclinical models (12); extension of this effect to CAR-T products is plausible but has not been demonstrated directly in published studies and should be treated as a hypothesis (6, 12). Sequencing matters: clearing NETs before or at the time of adoptive transfer maximizes effector entry, whereas NET formation during lymphodepletion conditioning can re-erect the barrier. Together with the exhaustion data in Section 4.4, these considerations support evaluation of NET- and PAR2-directed agents with checkpoint blockade and adoptive cell therapy, with trials stratifying patients by NET burden and PAR2 pathway activity (6, 12).

**Table 2** consolidates the intervention strategies discussed in this chapter, together with their principal models, observed effects, and current evidence level.

**Table 2:** Targeted intervention strategies against the NETs-PAR2 signaling axis.

Strategy/target	Representative agent(s)	Principal models	Observed effects	Evidence level
NET formation (PAD4)	Cl-amidine; BB-Cl-amidine	Murine breast cancer and colorectal liver metastasis models	Reduced NET burden and metastatic burden (8, 15); reduced liver metastatic burden via cancer-cell PAD4-driven ECM citrullination, a NET-independent mechanism (66)	Preclinical; BB-Cl-amidine in early clinical development
NET clearance	DNase I (free, nanoparticle-coated, hydrogel-delivered)	PDAC and breast cancer mouse models	Restored gemcitabine sensitivity; reduced lung metastasis (8, 23, 67); suppressed post-resection relapse (74)	Preclinical; DNase I entered early-phase trials as a chemotherapy adjunct
Upstream NETosis signaling	Sivelestat (NE inhibitor); DPI (NOX inhibitor); CXCR1/2 blockade	Colorectal and pancreatic cancer models	Reduced NET formation and metastasis; restored chemosensitivity (23, 69)	Preclinical
Natural products/repurposed drugs	Kaempferol; triptolide; icaritin; selinexor	Breast and urothelial cancer models	Inhibited NET formation and metastasis; proposed synergy with checkpoint inhibitors (70–73)	Preclinical, predominantly *in vitro*
PAR2 blockade (small molecules)	GB88 (biased); I-191 (pan-pathway); AZ8838 (intracellular allosteric)	NET-tumor co-culture; human colon and ovarian cancer cells	Inhibited NET-induced invasion; blocked calcium, ERK, RhoA, and β-arrestin signaling arms (11, 75, 77, 78)	Preclinical (tool compounds)
PAR2 blockade (antibodies)	Anti-PAR2 monoclonal antibody (mAb26685)	Xenograft models	Reduced primary-tumor invasion depth and lung/bone metastases (9)	Preclinical
Combination with immune checkpoint therapy	DNase I + anti-PD-1	Syngeneic murine tumors	Restored CD8+ T cell infiltration; reduced exhaustion markers (12); benefits for adoptive cell therapy inferred, not directly demonstrated	Preclinical
Combination with targeted therapy	PAR2 blockade + osimertinib	EGFR-mutant NSCLC cells	Reversed TKI resistance; reduced ERK-mediated EMT and PD-L1 expression (13)	Preclinical (*in vitro*)

NE, neutrophil elastase; NOX, NADPH oxidase; PDAC, pancreatic ductal adenocarcinoma; TKI, tyrosine kinase inhibitor; CAR-T, chimeric antigen receptor T cell.

## 8 Heterogeneous expression of NETs-PAR2 signaling in different tumor types

The intensity and functional consequences of NETs-PAR2 signaling vary substantially across tumor types, with differences in protease milieu, stromal architecture, and immune contexture. The same axis that drives dormant-cell reawakening and vascular adhesion in breast cancer operates through desmoplastic stromal reprogramming in pancreatic cancer, inflammatory bypass of targeted therapy in lung cancer, and dual pro- and anti-metastatic signaling in colorectal cancer. This heterogeneity is not noise; it defines which patients are likely to benefit from which intervention. **Figure 4** compares the axis across the four best-studied tumor types, and **Table 1** grades the strength of the supporting evidence layer by layer.

**Figure 4:**
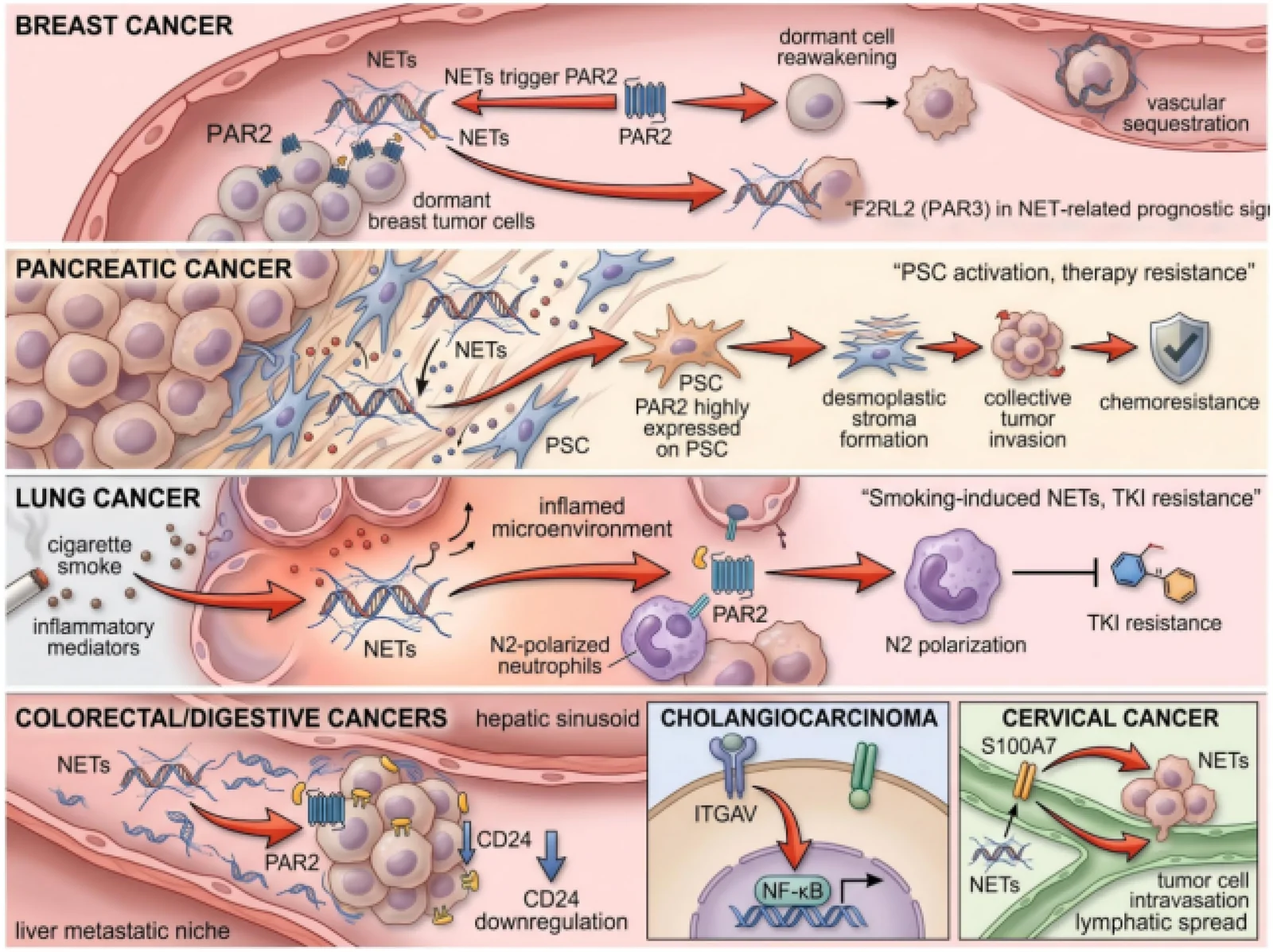
Comparative summary of the NETs-PAR2 axis across tumor types: breast (dormant-cell reawakening, vascular sequestration of circulating tumor cells, NET-related prognostic signature including F2RL2, note that F2RL2 encodes PAR3, a PAR2 paralog, and is not direct evidence for the axis), pancreatic (desmoplastic stroma, PSC activation, collective invasion, chemoresistance), lung (smoking-related inflammation, N2 polarization, TKI resistance), colorectal/digestive (liver metastatic niche, CD24 downregulation, cholangiocarcinoma ITGAV/NF-κB scaffold signaling, cervical cancer S100A7-NETs lymphatic spread). For each tumor: dominant PAR2-expressing cell type, key downstream effectors, and therapeutic implications.

### 8.1 Breast cancer

In triple-negative breast cancer (TNBC), the clearest link between NETs and recurrence runs through reactivation of dormant tumor cells. NETs produced during pulmonary inflammation awaken dormant cancer cells through protease-driven remodeling of the pericellular niche: NET-derived NE and MMP9 sequentially cleave laminin-111, exposing a cryptic epitope that activates integrin α3β1 and downstream FAK/ERK/MLCK/YAP signaling (16). This awakening mechanism is PAR2-independent, illustrating that NET proteases drive invasion through both PAR2-dependent and PAR2-independent routes; and chemotherapy itself induces lung NETs that perform the same awakening function, a mechanism with direct implications for the timing of adjuvant treatment (17). Metastatic breast cancer cells themselves induce neutrophils to form metastasis-supporting NETs *in vivo* (8), and circulating NETs can sequester tumor cells, promoting vascular arrest and embolization; this physical sequestration couples NET burden directly to the arrest step that determines whether circulating tumor cells can colonize distant capillaries (6).

Subtype-dependence is evident within breast cancer. In ER-positive tumors the axis appears less active than in TNBC, reflecting the less inflamed, less immune-suppressed microenvironment of hormone receptor-positive disease (6, 18). The PAR-family gene F2RL2 has been identified within a NET-related prognostic signature in breast cancer (18); however, F2RL2 encodes PAR3 rather than PAR2 itself (F2RL1), and that study did not functionally dissect the individual paralogs. We therefore do not count F2RL2 as clinical evidence for the NET-PAR2 axis; the breast cancer column of **Table 1** has been annotated accordingly. *In vitro*, NETs induce TF expression in breast cancer cells, and the TF/PAR2 axis mediates much of the NET-induced pro-tumor gene expression (9); *in vivo*, PAD4 inhibition and DNase I both reduce breast cancer metastasis, with DNase I-coated nanoparticles targeting the lung pre-metastatic niche among the best-controlled demonstrations of NET-directed therapy (8, 15). The breast cancer data form the most complete body of evidence connecting NETs, PAR2, and metastasis, yet derive almost exclusively from TNBC and basal-like models, a caveat tempering generalization to hormone receptor-positive disease.

### 8.2 Pancreatic cancer

Pancreatic ductal adenocarcinoma (PDAC) is defined by a desmoplastic, hypovascular stroma that impedes drug delivery and immune infiltration. NETs interact with PAR2 on pancreatic stellate cells (PSCs) to enhance collagen and fibronectin deposition, creating a rigid matrix that both impedes therapeutic agents and acts as a niche retaining NETs, perpetuating a feed-forward loop of PAR2 activation and ECM remodeling (19, 20). PAR2-mediated FAK activation favors collective invasion, matching the nested, cord-like histology of PDAC (6, 19). NETs also act on cancer cells through additional routes: the CCDC25-ITGB1-ILK axis promotes EMT and invasion (80), TIMP1 induces NET formation that promotes PDAC progression via CD63/ERK signaling (21, 81), tumor-derived NGAL reprograms cancer-associated fibroblasts through ITGA5 (19), and the GPRC5A-CXCL8-NET-cfDNA axis drives resistance to gemcitabine plus nab-paclitaxel (22).

The stromal barrier has direct therapeutic consequences. PAR2 antagonists face severe penetration problems in PDAC: systemic administration results in subtherapeutic intratumoral concentrations, and even effective antagonists may not reach the NET-rich invasive front. This has motivated local delivery and nanomedicine platforms, as well as combination strategies, such as co-targeting IL-17 and ITGA5 together with PD-1 blockade has been proposed for PDAC (19, 20). The PDAC experience shows that the same molecular axis can exert its effects through entirely different cell populations (stellate cells and fibroblasts rather than cancer cells alone), and that successful translation will require delivery science as much as receptor pharmacology.

These pro-invasive findings must be reconciled with reports of opposing PAR2 effects in pancreatic models. PAR2 activation suppressed lymphangiogenesis and lymph node metastasis in a murine pancreatic cancer model (57), even as PAR2 on stellate cells and fibroblasts promotes stromal invasion (19, 20). The contradiction likely reflects differences in the responding cell compartment (lymphatic endothelium versus cancer cells versus stellate cells), in local protease availability, and in downstream signaling bias, and it underlines that the pancreatic evidence for the axis is compartment-specific rather than uniform (Section 7.5).

### 8.3 Lung cancer

In non-small cell lung cancer (NSCLC), smoking-related chronic inflammation sustains neutrophil activation and NET formation. Elevated NET levels in the lung tumor tissue of smokers correlate with increased PAR2 expression and ERK phosphorylation in tumor cells, defining a NETs-PAR2-ERK axis that links smoking to invasion and metastasis (24). Tumor-associated neutrophils polarize toward a pro-tumorigenic N2 phenotype that maintains NETosis, secretes matrix remodeling factors, and suppresses antitumor immunity (24). This dual role makes the NETs-PAR2 axis an attractive target in lung cancer: intervening at PAR2 signaling could simultaneously reduce NET-driven invasion and dampen N2-mediated immune suppression.

The axis also intersects with targeted therapy resistance. In EGFR- or ALK-positive NSCLC, activation of NETs-PAR2 signaling can partially restore ERK and AKT phosphorylation despite tyrosine kinase inhibitor (TKI) treatment, a non-genetic bypass mechanism that reactivates the very survival signals TKIs suppress, without requiring new driver mutations. PAR2 blockade reverses osimertinib resistance in NSCLC cells by attenuating ERK-mediated EMT and PD-L1 expression, indicating that combining PAR2 antagonists with TKIs could delay or prevent resistance (13). Histological transformation of NSCLC to SCLC is an additional, independent mechanism of TKI resistance that PAR2-directed therapy is unlikely to address (82, 83); NETs-PAR2 blockade is therefore a rational partner for TKI-based regimens in EGFR/ALK-driven disease, but should not be expected to overcome resistance arising from lineage switching.

### 8.4 Colorectal cancer and other digestive system tumors

In colorectal cancer (CRC), NETs and PAR2 each contribute to the earliest stages of liver metastatic niche formation, and the evidence for their coupling is among the most instructive because it is bidirectional. NETs facilitate tumor cell adhesion and survival within hepatic sinusoids, and tumor cells exploit PAR2 to enhance adhesion to liver sinusoidal endothelial cells, promoting retention and evasion of immune clearance (11); single-cell transcriptomic analyses of CRC have begun to map which cell populations produce and respond to NETs within this niche (26). A striking counterpoint: NET-derived neutrophil elastase can inactivate canonical PAR2 signaling and thereby downregulate the phagocytic checkpoint CD24, promoting macrophage phagocytosis and inhibiting CRC liver metastasis (11). The same NETs-PAR2 machinery can thus either promote or suppress metastasis depending on the receptor’s signaling state and the dominant downstream readout, a central lesson showing that PAR2 is not a simple oncogenic switch, and that therapeutic interventions must preserve or restore the receptor’s suppressive functions while blocking its pro-invasive ones (see Section 7.5).

In gastric and esophageal cancers, environmental and infectious factors drive NETosis in the gastric mucosa. High-salt diets and Helicobacter pylori infection are potent inducers of gastric mucosal NET formation; elevated NET levels correlate with enhanced serosal infiltration of gastric cancer cells and increased peritoneal dissemination (reviewed in 84). The H. pylori connection is mechanistically coherent: the bacterium recruits neutrophils and provides both PAMPs and virulence factors that trigger NETosis, while chronic gastritis sustains a NET-rich inflammatory milieu for years (84); NET-related gastric carcinogenesis could therefore potentially be intercepted at the level of infection control, although direct evidence remains limited. In biliary tract cancers, NETs serve as a physical scaffold promoting cholangiocarcinoma progression through ITGAV/NF-κB signaling (27); in cervical cancer, S100A7-driven NET formation facilitates lymph node metastasis (28). Across these systems, NETs both provide a physical scaffold for adhesion and migration and engage PAR2-mediated signaling that enhances tumor cell survival, invasiveness, and immune evasion (11, 27, 28, 84).

### 8.5 When PAR2 signaling is protective: the counter-case

Four findings run against a uniformly pro-invasive axis and bear directly on the safety of PAR2 blockade, so we consolidate them here rather than scattering them as qualifications. First, PAR2 activation suppressed lymphangiogenesis and lymph node metastasis in a murine pancreatic cancer model (57), the opposite of its pro-angiogenic effect on blood vessels (Section 4.3). Second, in colorectal cancer, NET-derived neutrophil elastase downregulated the phagocytic checkpoint CD24 through PAR2 signaling, promoting macrophage phagocytosis and inhibiting liver metastasis (11). Third, PAR2 deficiency impaired antitumor immunity and attenuated anti-PD-1 efficacy in a colorectal cancer model (14). Fourth, in intestinal epithelial cells, F2RL1/PAR2 deficiency aggravated colitis-associated carcinogenesis through suppressed FOXA2/ATP6V0E1 and autophagy dysfunction, and neutrophil serine proteases acted on this pathway in a PAR2-dependent manner, identifying PAR2 as a protective receptor in the inflamed gut (41). Together, these cases show that the biological output of PAR2 is determined by the responding cell type, the protease milieu, and the tissue context; blanket chronic PAR2 blockade could be counterproductive in the gut and in tumors treated with checkpoint inhibitors, making tissue- and context-selective engagement a pharmacological requirement rather than a nicety (Sections 6.3 and 8.4).

## 9 Research prospects and unresolved issues

Several unresolved questions constrain progress on the NETs-PAR2 axis, falling into three categories. First, cellular heterogeneity: PAR2 signaling differs among tumor, immune, and stromal cells, and neutrophils polarize into N1 (anti-tumorigenic) and N2 (pro-tumorigenic) subsets; bulk analyses cannot resolve these contributions, and single-cell plus spatial multi-omics will be required to map intercellular communication *in situ*. Second, model fidelity: most studies rely on NETs generated ex vivo with PMA or bacterial stimuli, whose composition differs from intratumoral NETs; organoid-neutrophil co-cultures, TIME-on-chip platforms (47), and inducible, cell type-specific genetic models are needed to close this gap. Third, safety: NETs and PAR2 have essential physiological roles in host defense, tissue repair, and pain sensing, and chronic blockade could impair antimicrobial immunity; clinical development must therefore integrate infection surveillance and immune-competence biomarkers.

### 9.1 Dynamic tracking of the spatiotemporal evolution of NETs-PAR2 signaling

Real-time visualization of NETs-PAR2 signaling during invasion would substantially advance mechanistic understanding, because the temporal sequence of events is currently inferred from fixed-tissue snapshots rather than observed directly. Near-infrared fluorescent antibodies against citrullinated histone H3, combined with multiphoton intravital microscopy, allow non-invasive longitudinal tracking of NET formation in the native TME, and FRET-based PAR2 conformational sensors can distinguish transient from sustained receptor activation, a biologically meaningful distinction because transient and sustained signals engage different downstream programs (42). Integrating spatial transcriptomics with *in situ* proximity ligation assays could yield a “spatial pharmacodynamic map” of NETs-PAR2 activation across the invasion front (6, 37), and machine-learning approaches already used to identify NETs-related gene modules in multiple myeloma illustrate the analytical power of such workflows (54). These readouts would also provide pharmacodynamic endpoints for early-phase trials; technical barriers remain, including stable FRET sensors in hard-to-transfect primary cells and imaging depth limits in fibrotic tumors, but none is fundamental.

### 9.2 Post-translational modification regulation of the NETs-PAR2 signaling axis

Post-translational modifications (PTMs) constitute an under-explored regulatory layer of the axis. First, NET-associated proteases can alter PAR2 glycosylation: the receptor is heavily N-glycosylated, and glycosylation status affects ligand-binding affinity and G protein coupling bias, so deglycosylation could shift the balance between Gαq/11-dependent and β-arrestin-dependent signaling. Second, phosphorylation of the PAR2 C-terminal tail governs desensitization, β-arrestin recruitment, and signaling duration; NET stimulation may reshape this phosphorylation profile, but direct evidence linking NET-induced PTM changes to altered downstream signaling is largely absent. Third, NET-driven citrullination extends beyond histones: tumor cell proteins can be citrullinated by NET-derived PAD activity, potentially reshaping PAR2-dependent transcriptional programs (2). Site-specific PTM mapping in tumor models carrying point mutations at the modified residues is needed to determine whether any of these modifications represent druggable nodes. The PTM layer is attractive because it is modification-specific rather than receptor-wide: blocking a single pro-invasive phosphorylation event might preserve the receptor’s protective functions in host defense.

A parallel line of inquiry concerns histone modifications beyond citrullination. Histone lactylation, a metabolite-driven PTM that links glycolysis to gene regulation, regulates NET formation under inflammatory conditions, and its modulation by natural compounds can attenuate NET release *in vivo* (62). If lactylation and other metabolic PTMs prove to be general regulators of the NET-PAR2 loop, they would connect the metabolic state of the TME to axis activity and suggest additional intervention points, such as inhibitors of the enzymes that write or erase these marks (2, 62).

### 9.3 Clinical translational biomarker development

A clinically useful biomarker model for the NETs-PAR2 axis should integrate several layers of evidence rather than rely on any single analyte. Candidate components include NET markers (CitH3-DNA, NE-DNA), soluble PAR2 extracellular domain fragments released upon receptor cleavage, and active MMP9; a panel combining these would in principle capture both the burden of NETs and the degree of downstream pathway engagement.

The practical hurdles are as much pre-analytical as analytical. Circulating NET markers are exquisitely sensitive to sample handling: ex vivo neutrophil activation during phlebotomy can generate NETs in the tube, inflating CitH3-DNA measurements, and freeze-thaw cycles degrade the immune complexes that many assays detect; standardized protocols are therefore prerequisites for multicenter validation (6, 7). The soluble PAR2 ectodomain is an attractive analyte because its appearance in plasma reflects receptor activation in real time and is measurable with existing immunoassays; peripheral blood neutrophil surface PAR2 density is a complementary “liquid biopsy” surrogate measurable by flow cytometry (6). Transcriptomic signatures centered on EMT scores and MMP-family expression could further stratify patients by molecular invasion phenotype (18, 54). The critical bottleneck is validation: prospective multicenter cohorts with standardized pathological scoring and imaging correlation are required, and biomarker development should proceed in parallel with therapeutic development, because a pathway-activity biomarker is also the most reliable tool for selecting patients in whom NET/PAR2 blockade is likely to help rather than harm.

### 9.4 Bridging basic research to clinical translation

Translation of the NETs-PAR2 axis faces model-related and conceptual hurdles. Humanized mouse models that reconstruct both the human immune system and the TME are needed to evaluate the axis under conditions of adaptive immunity, since xenograft models miss the immune interactions that dominate in patients (6, 9, 11); organoid and multi-organ-chip platforms offer controlled systems for mechanistic validation and drug screening (6, 47). On the conceptual side, the field must confront the context-dependence of PAR2 signaling: PAR2 deficiency can impair antitumor immunity and attenuate anti-PD-1 efficacy in colorectal cancer (14), and NET-derived elastase can downregulate CD24 to promote macrophage-mediated clearance of CRC liver metastasis (11). Blanket inhibition of the axis could therefore be counterproductive in some patients, and biomarker-guided, context-aware trial design will be essential. Combination strategies are already being proposed: PAR2 antagonists with cetuximab for advanced colorectal cancer (6, 11), NET clearance with immune checkpoint blockade (12), and PAD4 or DNase I-based approaches layered onto chemotherapy in PDAC (19, 23). A realistic first-generation trial would be a biomarker-enriched phase II study in a tumor type with strong preclinical support (e.g., EGFR-mutant NSCLC or TNBC), randomizing patients with high NET burden to standard therapy with or without a NET/PAR2-directed agent, with correlative sampling to validate the pharmacodynamic assay. The axis is unlikely to yield a single blockbuster target; its value lies in the precision with which it can be engaged in patients where the balance of evidence is pro-invasive (6, 11, 12).

## 10 Discussion

The NETs-PAR2 signaling axis links inflammation, immune regulation, and tumor progression. The survey presented here supports a conclusion that is narrower than the vocabulary of the field sometimes implies: findings concerning NETs alone (for example, T cell entrapment and exhaustion) or PAR2 alone (for example, bypass of TKI resistance) are far more abundant than findings in which both components are directly connected. The strongest direct links remain the TF/PAR2-dependent response of breast cancer cells to NET stimulation and the PAR2-dependent, PAD4-independent route of mtDNA-NET formation; most other nominally axis-level findings are converging but indirect, and we have graded every mechanistic claim accordingly (strong, moderate, emerging).

The heterogeneity of these effects demands equal attention. The intensity and functional outcome of PAR2 activation vary with tumor genetics, stromal composition, hypoxia, immune context, and the responding cell type; PAR2 can promote invasion in one setting, support productive antitumor immunity in another (14), or inhibit lymph node metastasis in a pancreatic model (57). Subtype-specific and context-aware strategies are therefore more realistic than a one-size-fits-all approach, and they will require biomarkers that report which PAR2 signaling mode predominates in a given patient.

A pragmatic reading of the current evidence suggests where the axis is most likely to be actionable first. The strongest experimental support exists in triple-negative breast cancer, where NET clearance by DNase I or PAD4 inhibition reproducibly reduces metastasis across independent laboratories (8, 15); in EGFR-mutant lung cancer, where PAR2 blockade reverses resistance to a drug already in routine clinical use (13); and in colorectal liver metastasis, where the PAR2-CD24 axis directly controls the seeding of the metastatic niche (11). The counter-case assembled in Section 7.5 defines the safety envelope within which such interventions should be developed: patients with high gut inflammatory burden, or tumors dependent on productive PAR2 signaling for immune surveillance, are precisely those in whom chronic blockade may harm. The pancreatic cancer case remains the most challenging, because the desmoplastic barrier limits drug access, but it is also where the unmet need is greatest, and local or nanomedicine-based delivery may ultimately make the axis druggable there (19, 20). How these considerations translate into trial design, biomarker development, and model systems is detailed in Section 8; here it suffices to note that the tools required to convert correlative into causal claims (intravital imaging, proximity ligation, cell type-specific genetics) already exist. **Figure 5** recapitulates the chain of evidence and the therapeutic entry points discussed in this review.

**Figure 5:**
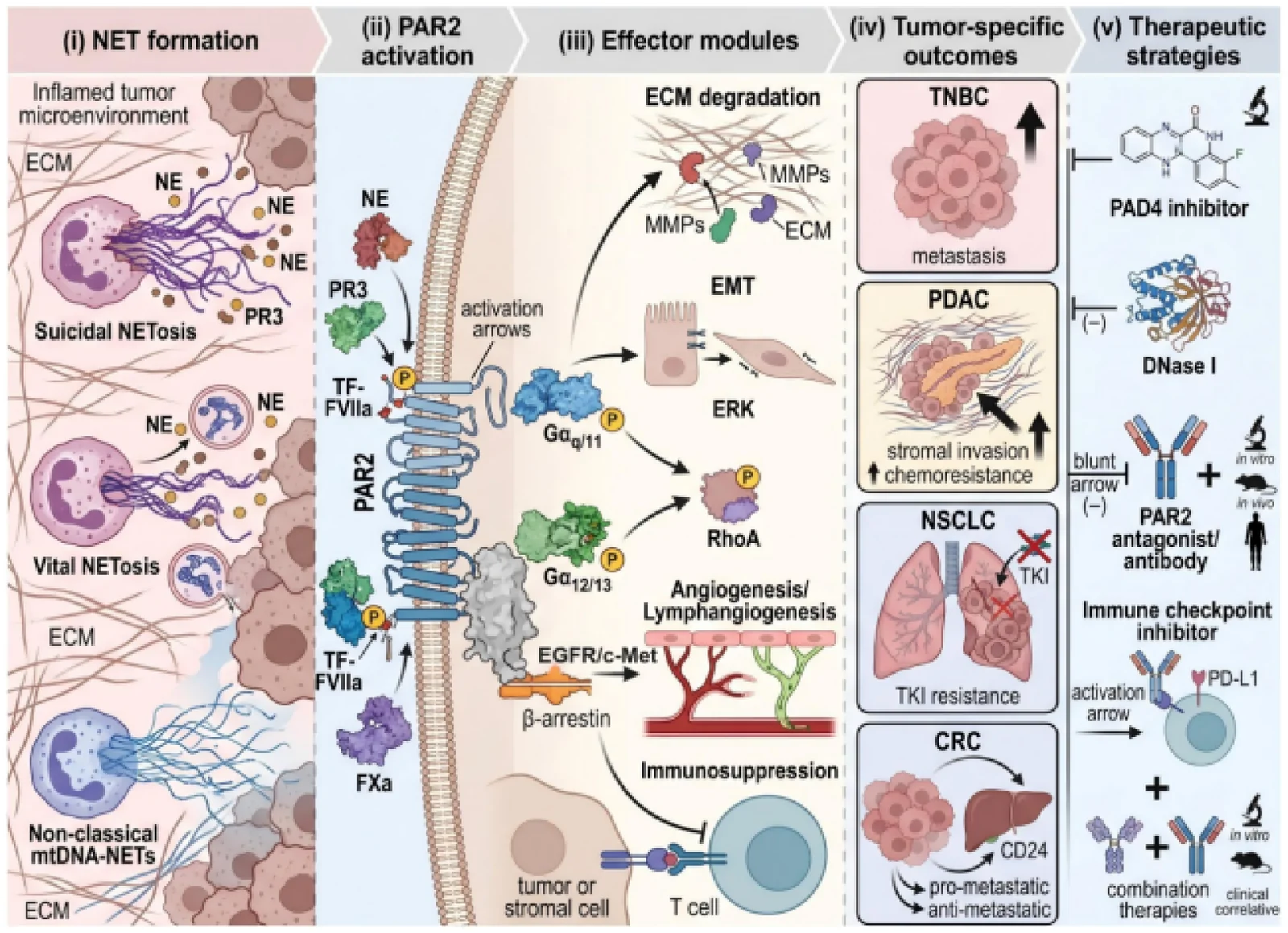
Summary graphic of the whole review. Left to right: (i) NET formation: suicidal and vital NETosis plus non-classical mtDNA-NETs in the inflamed tumor microenvironment; (ii) PAR2 activation: cleavage by NET-derived NE/PR3 and coagulation proteases (TF-FVIIa, FXa), biased signaling via Gαq/11-ERK, Gα12/13-RhoA, β-arrestin, and EGFR/c-Met transactivation; (iii) four effector modules: ECM degradation, EMT, angiogenesis/lymphangiogenesis, immunosuppression; (iv) tumor-type-specific outcomes: TNBC metastasis, PDAC stromal invasion and chemoresistance, NSCLC TKI resistance, CRC liver metastasis with CD24-dependent dual effects; (v) therapeutic entry points: NET inhibition (PAD4 inhibitors, DNase I), PAR2 antagonists/antibodies, and combinations with immune checkpoint blockade, each annotated with the evidence tier (*in vitro*, *in vivo*, clinical correlative) supporting it.

## Glossary

ALK: Anaplastic lymphoma kinase
Ang-2: Angiopoietin-2
APC: Adenomatous polyposis coli
CAF: Cancer-associated fibroblast
CAR-T: Chimeric antigen receptor T cell
CCR: C-C chemokine receptor
CitH3: Citrullinated histone H3
CRC: Colorectal cancer
CTC: Circulating tumor cell
CXCL: C-X-C motif chemokine ligand
CXCR: C-X-C chemokine receptor
DAMP: Damage-associated molecular pattern
DNase I: Deoxyribonuclease I
DPI: Diphenyleneiodonium
ECM: Extracellular matrix
EGF: Epidermal growth factor
EGFR: Epidermal growth factor receptor
EMT: Epithelial-mesenchymal transition
ERK: Extracellular signal-regulated kinase
FAK: Focal adhesion kinase
FGF: Fibroblast growth factor
FRET: Förster resonance energy transfer
GPCR: G protein-coupled receptor
HCC: Hepatocellular carcinoma
HGF: Hepatocyte growth factor
HIF-1α: Hypoxia-inducible factor 1-alpha
HMGB1: High-mobility group box 1
IL: Interleukin
JNK: c-Jun N-terminal kinase
LAG-3: Lymphocyte-activation gene 3
MDSC: Myeloid-derived suppressor cell
MMP: Matrix metalloproteinase
MPO: Myeloperoxidase
mtDNA: Mitochondrial DNA
NE: Neutrophil elastase
NETs: Neutrophil extracellular traps
NF-κB: Nuclear factor kappa B
NSCLC: Non-small cell lung cancer
PAD4: Peptidylarginine deiminase 4
PD-1: Programmed cell death protein 1
PD-L1: Programmed death-ligand 1
PDAC: Pancreatic ductal adenocarcinoma
PGE2: Prostaglandin E2
PKC: Protein kinase C
PLCβ: Phospholipase C beta
PR3: Proteinase 3
PROTAC: Proteolysis-targeting chimera
PSC: Pancreatic stellate cell
RhoA: Ras homolog family member A
ROCK: Rho-associated kinase
ROS: Reactive oxygen species
RTK: Receptor tyrosine kinase
SCLC: Small cell lung cancer
TAM: Tumor-associated macrophage
TF: Tissue factor
TGF-β: Transforming growth factor beta
TLR: Toll-like receptor
TME: Tumor microenvironment
TNBC: Triple-negative breast cancer
uPA: Urokinase-type plasminogen activator
VEGF: Vascular endothelial growth factor
VEGFR: Vascular endothelial growth factor receptor
ZEB: Zinc finger E-box-binding homeobox.
